# Self‐assembly and Lateral Cobalt Coordination of a Fivefold Symmetric Cyanostar Macrocycle on an Au(111) Surface

**DOI:** 10.1002/chem.202503051

**Published:** 2025-12-03

**Authors:** Lenka Černá, Miguel Martínez García, Shanmugasibi K. Mathialagan, Sofía O. Parreiras, Koen Lauwaet, José Ignacio Urgel, Aurelio Gallardo, Amar H. Flood, Tomás Torres, José M. Gallego, Giovanni Bottari, David Écija

**Affiliations:** ^1^ Instituto Madrileño de Estudios Avanzados en Nanociencia (IMDEA Nanoscience) Madrid Spain; ^2^ Departamento de Química Orgánica Universidad Autónoma de Madrid Madrid Spain; ^3^ Unidad de Nanomateriales Avanzados IMDEA Nanoscience, Unidad asociada al CSIC por el ICMM Madrid Spain; ^4^ Department of Chemistry Indiana University Bloomington Indiana USA; ^5^ Institute for Advanced Research in Chemical Sciences (IAdChem) Universidad Autónoma de Madrid Madrid Spain; ^6^ Instituto de Ciencia de Materiales de Madrid (ICMM) CSIC Madrid Spain

**Keywords:** cyanostar, metal‐ligand coordination, on‐surface chemistry, scanning tunneling microscopy, self‐assembly

## Abstract

The functionalization of solid surfaces with responsive macrocyclic compounds is a key strategy for developing advanced functional materials, with applications in molecular sensing, catalysis, and nanoscale electronics. Here, we report the self‐assembly and lateral cobalt coordination, on an Au(111) surface and under ultra‐high vacuum conditions, of a fivefold symmetric cyanostar macrocycle, a class of anion recognition molecules. This represents the first example of a close‐packed regular assembly of a pentagonal macrocycle at the solid‐vacuum interface.

## Graphical abstract

Herein, we report the first example of the formation of a close‐packed, regular 2D monolayer of a fivefold symmetric cyanostar macrocycle on an Au(111) surface under ultra‐high vacuum and the structural modification of such self‐assembled architecture upon cobalt atom deposition.

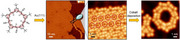

## 1 Introduction

The realization of large‐scale patterns of functional organic molecules on solid surfaces is a cornerstone of modern materials science, driving advances in catalysis, sensing, and molecular electronics [1, 2, 3, 4]. Among the available building blocks used for such purposes, macrocyclic compounds are particularly interesting since their pre‐organized cavities can be further leveraged to recognize and template specific guests [5, 6, 7, 8]. In this context, many works have appeared in the past reporting the formation of surface‐supported 2D arrays featuring macrocycles with different geometries and symmetries, both at the solid‐liquid interface [9, 10, 11, 12, 13] and in ultra‐high vacuum conditions [14, 15, 16, 17, 18, 19]. However, significantly scarcer are the reports dealing with fivefold symmetric macrocycles, which have been mostly investigated at the solid‐liquid interface [20, 21, 22]. To the best of our knowledge, the only systematic studies of close‐packing assemblies using pentagonal molecules at the solid‐vacuum interface have focused primarily on corannulene and some pentasubstituted derivatives [23, 24].

The scarcity of fivefold symmetric assemblies stems from a fundamental geometric issue: The *C*_5_ symmetry of a molecule is incompatible with the translational symmetry required for periodic tiling in 2D. None of the 17 plane groups that describe periodic tessellations permits fivefold rotations [25]. Consequently, while maximizing molecule‐substrate and molecule‐molecule interactions typically favors compact and high‐symmetry packing, pentagonal molecules cannot form a close‐packed, gapless monolayer, which implies that *C*_5_‐symmetric molecules are forced to form lower‐symmetry or incommensurate structures in order to achieve dense packing.

**FIGURE 1:**
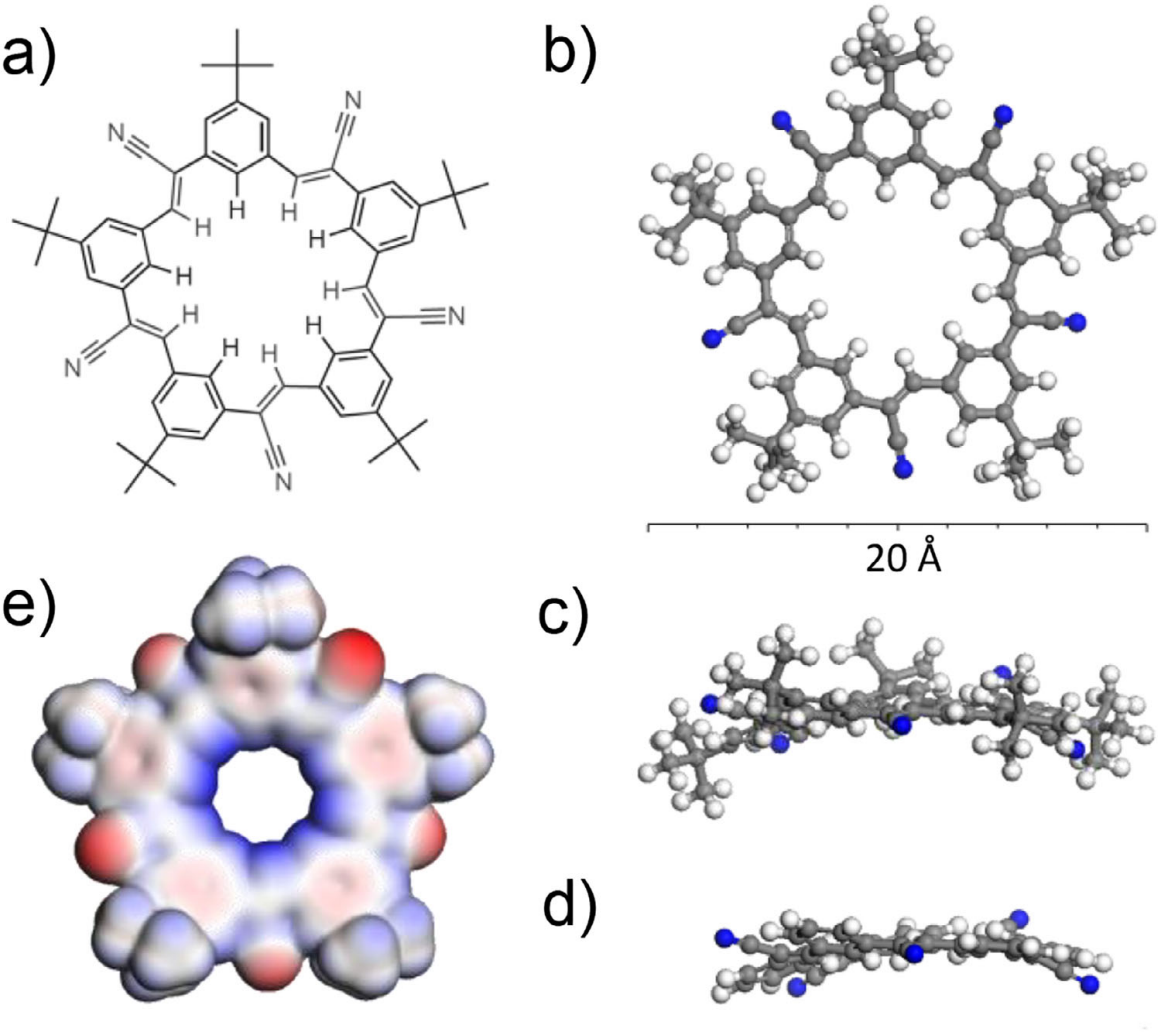
a) Chemical structure of cyanostar CS‐**1**. b) Top and c) side view of the DFT optimized geometry of CS‐**1** in the gas phase. d) Side‐view of an analogue of CS‐**1** where the five *tert*‐butyl groups have been replaced by hydrogen atoms to better see the tilting of the ‐CN groups. e) Electron density isosurface (0.02 electrons/Å^3^) colored according to the electrostatic potential at every point of the surface. The color scale goes from ‒1.69 eV/*e* (red) to 1.96 eV/*e* (blue), where *e* is the electron charge, indicating the electropositive character of the inner cavity, responsible for the molecular anion recognition capacity. In b), c), and d), carbon atoms are colored in gray, nitrogen atoms in blue, and hydrogen atoms in white.

Here, we address this issue by investigating CS‐**1**, a molecule which belongs to the family of *C*_5_‐symmetric macrocycles called cyanostars (Figure 1a) [26, 27]. The electron‐withdrawing nature of the five peripheral nitrile groups contributes to create an electropositive cavity that imparts to these macrocycles a strong affinity for large and charge‐diffuse anions, like ClO_4_^−^, BF_4_^−^ or PF_6_^−^ (Figure 1e) [22].

Furthermore, cyanostars can easily form π‐stacked 2:1 “sandwich” complexes thanks to their bowl shape along with the π−π interactions between the two molecules. These macrocycles can also stabilize organic radical anions [28] as well as dimers of phosphate anions, forming complexes with a 2:2 stoichiometry [29]. In particular, they can bind phosphates and phosphonates, enabling oligomerization and supramolecular polymerization [27, 30]. Cyanostars have found applications as novel battery electrolytes [31], anion‐selective sensors [27, 32], tunable adhesives [33], ultra‐bright fluorescence materials [34], or as potential photovoltaic materials [35].

Although several macrocycles adsorbed on solid surfaces have been reported to date [9, 10, 13, 36, 37], to the best of our knowledge, the only attempt at surface functionalization using a cyanostar was undertaken at the liquid‐solid interface employing an ester‐terminated derivative bearing *n*‐octyl‐functionalized phenylene‐acetylene arms in a solution of 1,2,4‐trichlorobenzene (TCB) on HOPG. This macrocycle gave rise to the formation of a 2D architecture in the form of pairs of anti‐parallel rows stabilized by attraction interactions between the ester units at the cyanostar's peripheral positions [22]. Interestingly, a different ordered arrangement, presumably of sandwich complexes, was also observed when a solution of PF_6_^‒^ was added to the CS/TCB solution [22].

Herein, we demonstrate that cyanostar CS‐**1** can be deposited under ultra‐high vacuum conditions on Au(111), yielding highly ordered monolayers, directly visualized by scanning tunneling microscopy (STM). Interestingly, significant modification of the cyanostar patterning was observed upon deposition of cobalt atoms because of the macrocycles lateral coordination by CN···Co···NC metal‐ligand interactions.

## 2 Results and Discussion

**FIGURE 2:**
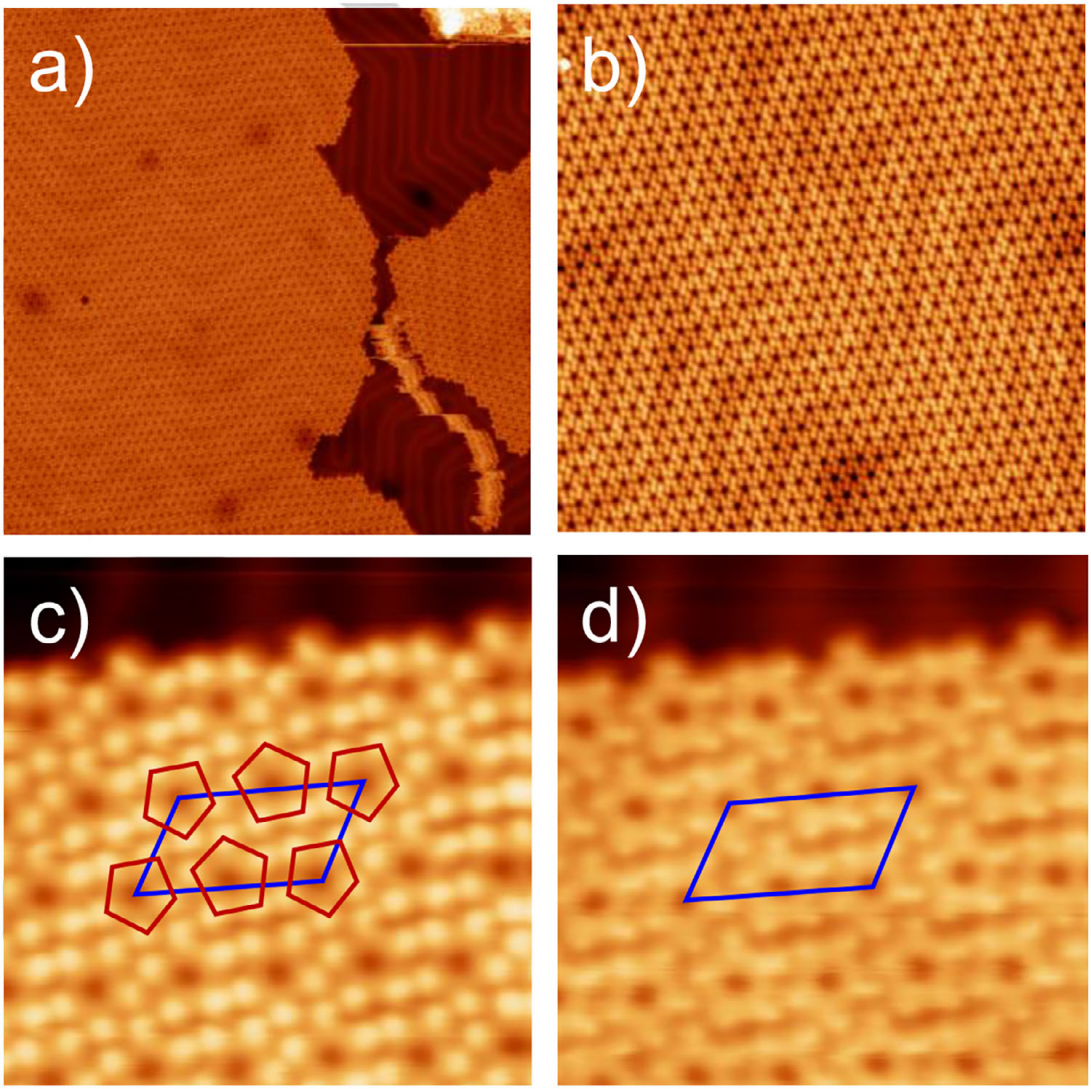
STM images of a submonolayer of CS‐**1** deposited on a Au(111) surface. a) 100 mV, 10 pA, 100 nm × 100 nm; b) 1 V, 10 pA, 50 nm × 50 nm; c) −100 mV, 10 pA, 10 nm × 10 nm; d) −10 mV, 10 pA, 10 nm × 10 nm. The blue rhomboids in (c) and (d) indicate the unit cell of the self‐assembly. In c) CS‐**1** appears as five round protuberances surrounding a central void. In d), recording the STM at lower bias voltages, the fivefold symmetric molecular shape of CS‐**1** is clearly visible.

Figure 2 shows STM images of the Au(111) surface after depositing a submonolayer coverage of CS‐**1** with the substrate held at room temperature. The high thermal stability of CS‐**1** [29] enables its sublimation at 315 °C. CS‐**1** self‐assembles on Au(111) to form large islands (Figure 2a) with an approximate hexagonal symmetry (Figure 2b). A closer look reveals that the unit cell is oblique (a = 18.9 Å, b = 35.0 Å, α = 117.0°) featuring two molecules per unit cell (Figure 2c). Under these conditions, the CS‐**1** macrocycles are imaged as five lobes around a central void (Figure 2c), although for certain voltages/tip conditions the star‐shaped molecular structure can be easily recognized (Figure 2d). Along the direction of the shorter side of the unit cell all the molecules are oriented pointing in the same direction, while along the longer side neighboring molecules are oriented in opposite directions (Figure 2c). Height profiles (Figure S2) and manipulation experiments (Figure S3) indicate that the islands are only one‐molecule high. Figures 2a,b also show that the gold herringbone reconstruction remains apparently intact upon the cyanostar adsorption, indicating a rather weak molecule‐substrate interaction.

It is interesting to notice that the assembly pattern of the Au(111)‐supported cyanostar CS‐**1** is very similar to that found in 2D cuts of bulk crystals of CS‐**1** [26], or a closely related *C*_5_‐symmetric macrocycle [38]. Prior works on the monolayer tiling of *C*_5_‐symmetric molecules studied using STM have shown the formation of various 2D patterns [20, 39]. All of them seem to follow one of the structures predicted by different theoretical works, the one formed by CS‐**1** corresponding to the so‐called anti‐parallel phase, which presents the highest packing density [40, 41, 42].

**FIGURE 3:**
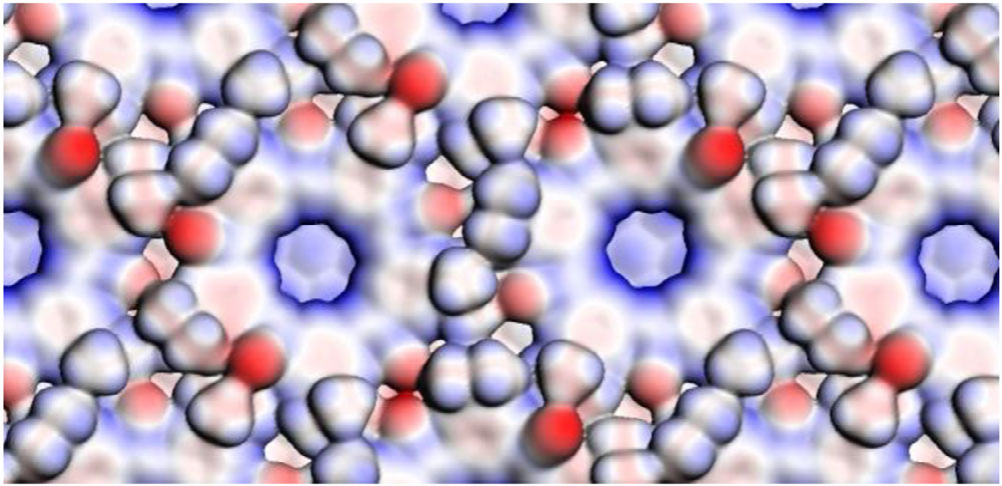
DFT calculated electronic isosurface (0.02 electrons/Å^3^) at two neighboring CS‐**1** molecules, colored according to the electrostatic potential at every point of the surface. The color scale goes from ‒1.32 eV/*e* (red) to 2.19 eV/*e* (blue), where *e* is the electron charge, showing that the inner cavity maintains its electropositive character after adsorption on the Au surface.

The STM data are compatible with a lattice with an epitaxial relationship with the Au substrate described by the matrix

$$\left(\begin{array}{cc} 7 & 1\\ -2 & 12 \end{array}\right)$$

with dimensions a = 18.9 Å, b = 37.8 Å, α = 120.0°, very close to the experimental ones (*vide supra*). The DFT optimized geometry of the self‐assembly of CS‐**1** on Au(111) based on this structure is shown in Figure S4. For comparison, Figure 3 displays an electronic isosurface colored according to the electrostatic potential, showing that the self‐assembly of CS‐**1** is based on a combination of van der Waals forces and electrostatic interactions between the electronegative cyano groups and the electropositive *tert*‐butyl groups. It is worth mentioning that, as occurring in the gas phase, the inner cavity of the cyanostar maintains its electropositive character even upon adsorption on the Au surface. This feature could potentially be used for anion post‐functionalization of these macrocycles, thus leading to the realization of ordered arrays of anions on the surface.

Although CS‐**1** is a pro‐chiral molecule [26] (*i.e*., chiral when adsorbed on a surface), the recorded STM images do not allow to distinguish such a chiral feature. DFT calculations, however, estimate that a homochiral assembly is only ∼0.13 eV per unit cell more stable than the one where the two molecules in the unit cell have opposite chirality, making the formation of random heterochiral assemblies a reasonable assumption. Consistent with this idea, in the 3D crystal structure of CS‐**1** [26], macrocycles of either chirality are found on identical lattice sites and are indistinguishable from each other using X‐ray diffraction.

Figure S5 shows the top and side views of the conformation of CS‐**1** within the self‐assembled pattern or adsorbed isolated on the Au(111) surface. Although shape persistent, CS‐**1** shows a large degree of flexibility, with calculations suggesting the presence, in solution, of over 332 conformers within a very small energy window between them [43]. In CS‐**1,** besides the *tert*‐butyl groups, most of the macrocycle flexibility comes from the local tilt of the ‐CN groups (which depends on the dihedral angles between the olefins and their neighboring phenylene groups). Thus, CS‐**1** is not perfectly flat in the gas phase (Figure 1b,c) but instead can access various conformations. In each of these, the cyano‐olefin groups tilt either above or below the macrocycle's mean plane, giving the molecule a ruffled semi‐planar geometry. The most stable conformations have two nonneighboring olefins tilted above the plane and three below (Figure 1c,d). When adsorbed isolated on the Au(111) surface (Figure S5b), except for the *tert*‐butyl groups, the molecule is almost flat, with the ‐CN groups forming angles between 1° and 2° with respect to the surface. In this configuration, the binding energy is 7.8 eV. On‐surface self‐assembly changes the molecular conformation (Figure S5a). Although the inner core is still mostly flat, some of the cyano groups tilt upwards, probably to maximize the interaction with the *tert*‐butyl groups of neighboring molecules.

**FIGURE 4:**
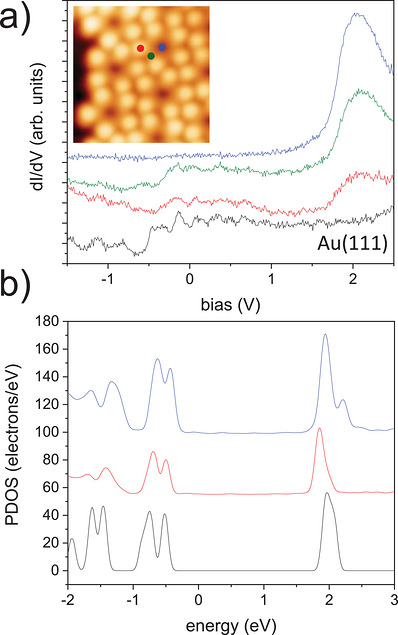
a) dI/dV spectra taken at different positions of a CS‐**1** molecule within an island. b) Projected density of states (PDOS) of CS‐**1** in gas phase, assuming a vacuum level alignment (black line), an isolated CS‐**1** molecule on Au(111) (red line), and a self‐assembled monolayer of CS‐**1** (blue line).

Scanning tunneling spectroscopy shows that the self‐assembled structure has a strong resonance around 2.0 eV (*i.e*., LUMO), but no clear features are visible at negative voltages (Figure 4a), possibly due to the exponential behavior of the transmission coefficient with the bias voltage [44]. Figure 4b shows the DFT calculated density of states of CS‐**1** in the gas‐phase assuming a vacuum level alignment [45] (black line), the projected density of states of an isolated CS‐**1** molecule adsorbed on the Au(111) surface (red line), and a self‐assembled monolayer on Au(111) (blue line). For the isolated molecule on gold (red line), the calculations place the LUMO at 1.85 eV, and the HOMO at −0.50 eV, to give a band gap of 2.35 eV, which is only slightly smaller than the gas‐phase HOMO‐LUMO separation of 2.49 eV (black line), indicating a rather weak interaction with the gold substrate. It is worth noticing that the experimental HOMO‐LUMO gap of CS‐**1** measured in solution is 3.45 eV [25, 33], a discrepancy that can be explained by considering that DFT usually underestimates the band gap [46]. The formation of the 2D assembly shifts the whole spectrum (blue line) to higher binding energies by ∼0.07 eV, probably due to a smaller charge transfer with the substrate. In this case, the LUMO appears at 1.94 eV, very close to the experimental results.

**FIGURE 5:**
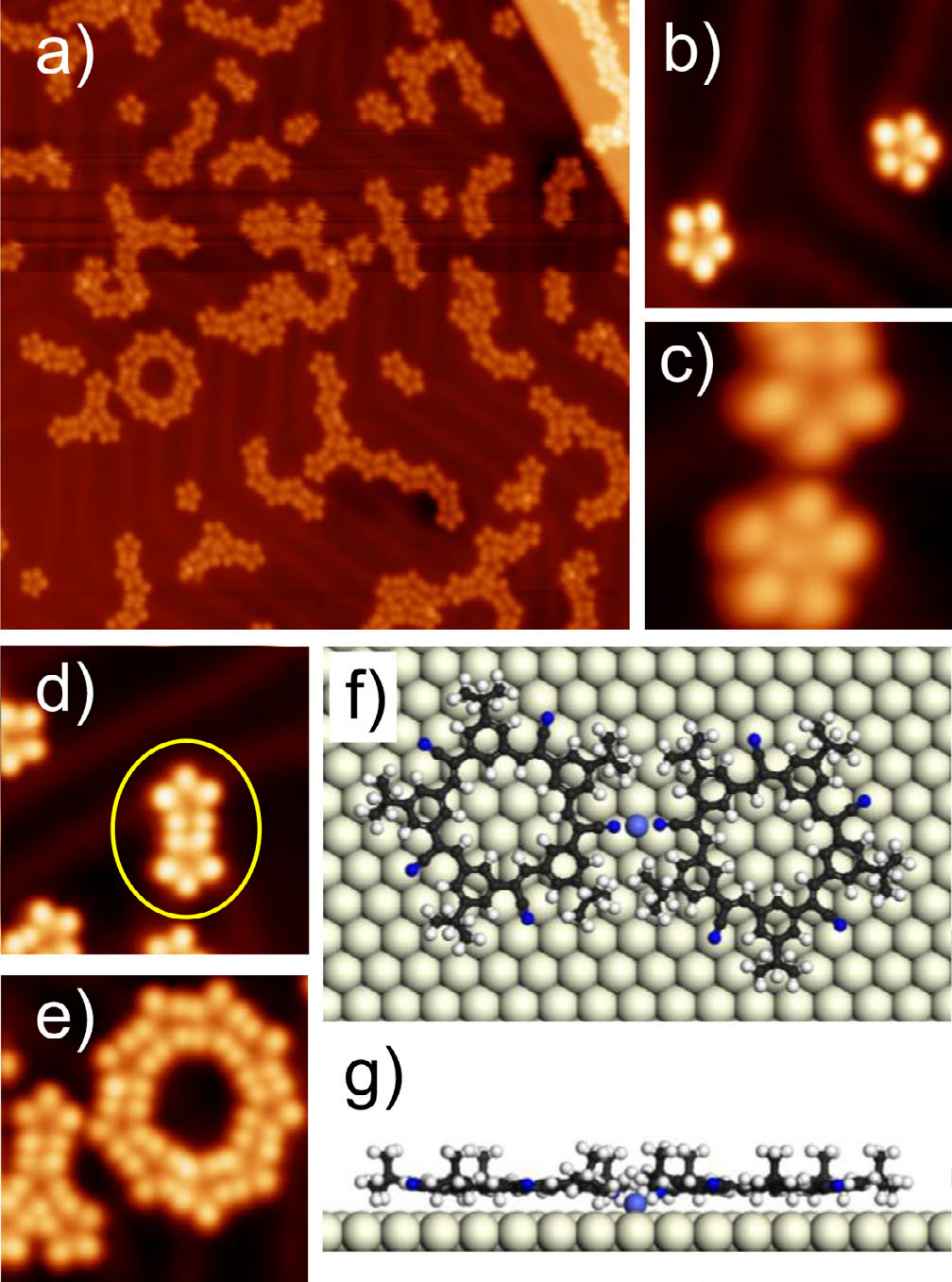
a‐e) STM images taken after depositing a small amount of Co atoms onto the self‐assembled CS‐**1** monolayer shown in Figure 2a. a) General overview, showing the different types of structures formed. b‐c) STM images of isolated molecules, some of them containing Co atoms embedded in the inner cavity. d) STM image of the basic structure responsible for the formation of the chains. e) STM image of a circular supramolecular ensemble composed of 9 cyanostar molecules coordinated through Co atoms. f) Top and g) side views of the DFT optimized structure of a (CS‐**1**)···Co···(CS‐**1**) complex on the Au(111) surface. a) 100 mV, 10 pA, 50 nm × 50 nm; b) 50 mV, 10 pA, 10 nm × 10 nm; c) 200 mV, 20 pA, 5 nm × 5 nm; d) 100 mV, 10 pA, 10 nm × 10 nm; e) 100 mV, 50 pA, 10 nm × 10 nm.

Following our interest in macrocycles metalation on surfaces [15, 47, 48] and the unexplored potential of cyanostars in this respect, we evaluated the capability of CS‐**1** to complex metal atoms in its central cavity by depositing a small amount of cobalt atoms on the self‐assembled 2D pattern of CS‐**1**. As Figure 5a shows, upon this treatment, the self‐assembled islands have disappeared, and a number of different motifs are now present on the surface, including isolated molecules, short branched oligomers, and even circular nanostructures. Remarkably, a few cyanostars featuring one or several bright spots in their inner cavity are also observed (*i.e*., less than 5%), probably due to the complexation of Co atoms (Figure 5b,c).

Concerning the oligomeric species, which relative abundance increased upon increasing the amount of Co atoms deposited, we hypothesize that their formation is the result of cobalt‐coordinated cyanostar macrocycles [49, 50]. The basic motif is highlighted in Figure 5d (yellow oval), which geometry suggests that the cyano groups of the two neighboring macrocycles face each other, a configuration energetically unfavorable unless stabilized by the presence of a Co atom coordinating the two CN groups. Top and side views of the DFT optimized geometry of such (CS‐**1**)···Co···(CS‐**1**) metalo‐organic complex are shown in Figure 5f,g. Importantly, the formation of a 2D extended coordination network is probably disfavored due to steric hindrance. In addition, some peculiar metal‐organic architectures are detected, as the circular assembly depicted in Figure 5e, comprising 9 cyanostars coordinated through Co atoms (Figure S6).

Considering that cyano groups can coordinate to many different metal ions (*e.g*., Ag, Cu, Fe, Pd, Na) [51], in principle, different cyanostar‐based metal‐organic structures could be obtained by replacing the Co atoms with any of these elements.

## 3 Conclusion

Herein, we have shown that the fivefold symmetric cyanostar macrocycle CS‐**1** self‐assembles on the Au(111) surface by forming large, highly ordered 2D arrays with an oblique unit cell, similar to the 2D packing the macrocycle forms in solid state, while retaining an electropositive cavity. Upon cobalt deposition, the formation of metal‐organic nanoarchitectures such as branched polymers and discrete circular supramolecules is observed. Future studies will explore the deposition of other atoms/ions on these cyanostar assemblies with the aim of fabricating patterned surfaces, unlocking new applications that leverage nanoscale ordering on electrodes.

## Conflicts of Interest

The authors declare no competing financial interest.

## Supporting information




**Supporting File 1**: chem70537‐sup‐0001‐SuppMat.pdf

## Data Availability

The data that support the findings of this study are available in the supplementary material of this article.
