# Supplementary material for: Self‐assembly and Lateral Cobalt Coordination of a Fivefold Symmetric Cyanostar Macrocycle on an Au(111) Surface
Source: Chemistry. 2025 Dec 3;32(24):e03051. doi: 10.1002/chem.202503051 (PMC13290421; doi:10.1002/chem.202503051)
Supplement: Supplementary file 1 — Supporting File 1: chem70537‐sup‐0001‐SuppMat.pdf [file CHEM-32-e03051-s001.pdf]

Supporting Information for

Self-assembly and Lateral Cobalt Coordination of a Five-fold Symmetric Cyanostar Macrocycle on a Au(111) Surface

Lenka Cerna,[a] Miguel Martínez García,[a,b] Shanmugasibi K. Mathialagan,[a] Sofía O. Parreiras,[a] Koen Lauwaet,[a] José Ignacio Urgel,[a,c] Aurelio Gallardo,[a] Amar H. Flood,[d] Tomás Torres,*[a,b,e] José M. Gallego,*[f] Giovanni Bottari,*[a,b,e] and David Écija*[a,c]

[a] Prof. D. Écija, Dr. G. Bottari, Prof. T. Torres, Dr. M. Martínez García, L. Cerna, S. K. Mathialagan, Dr. S. O. Parreiras, Dr. K. Lauwaet, Dr. J. I. Urgel, Dr. A. Gallardo
Instituto Madrileño de Estudios Avanzados en Nanociencia (IMDEA Nanoscience), 28049, Madrid, Spain

[b] Dr. M. Martínez García, Prof. T. Torres, Dr. G. Bottari
Departamento de Química Orgánica, Universidad Autónoma de Madrid, Campus de Cantoblanco, 28049 Madrid, Spain

[c] Dr. J. I. Urgel, Prof. D. Écija
Unidad de Nanomateriales Avanzados, IMDEA Nanoscience, Unidad asociada al CSIC por el ICMM, Madrid 28049, Spain.

[d] Prof. A. H. Flood
Department of Chemistry, Indiana University, Bloomington, Indiana 47405, United States of America

[e] Prof. T. Torres, Dr. G. Bottari
Institute for Advanced Research in Chemical Sciences (IAdChem), Universidad Autónoma de Madrid, 28049 Madrid, Spain

[f] Dr. J. M. Gallego
Instituto de Ciencia de Materiales de Madrid (ICMM), CSIC, 28049, Madrid, Spain

E-mail: giovanni.bottari@uam.es; josemaria.gallego@csic.es; tomas.torres@uam.es; david.ecija@imdea.org

Table of contents

List of abbreviations S2
1. Organic synthesis: Materials and Methods S3
2. Solution-based synthesis and characterization of CS-**1** and its precursors S3
3. Experimental methods and computational details S6
References S10

List of abbreviations

AcOEt = Ethyl acetate

DCM = Dichloromethane

EtOH = Ethanol

LAH = Lithium aluminium hydride

MeOH = Methanol

NMR = Nuclear magnetic resonance

PCC = Pyridinium chlorochromate

rt = Room temperature

THF = Tetrahydrofuran

TLC = Thin layer chromatography

1. Organic synthesis: Materials and Methods

Chemicals and solvents were purchased from commercial suppliers (Aldrich, BLDpharm, Thermo Scientific Chemicals and Scharlab) and used without further purification. All dry solvents were freshly distilled under argon over an appropriate drying agent before use. Column chromatography was carried out on silica gel VWR-60 (40-63 μm). Analytical TLC was performed on aluminum sheets precoated with silica gel 60 F-254 from Merck. ^{1}H- and ^{13}C-NMR spectra were recorded with a Bruker DPX 400 MHz instrument.

2. Solution-based synthesis and characterization of CS-1 and its precursors

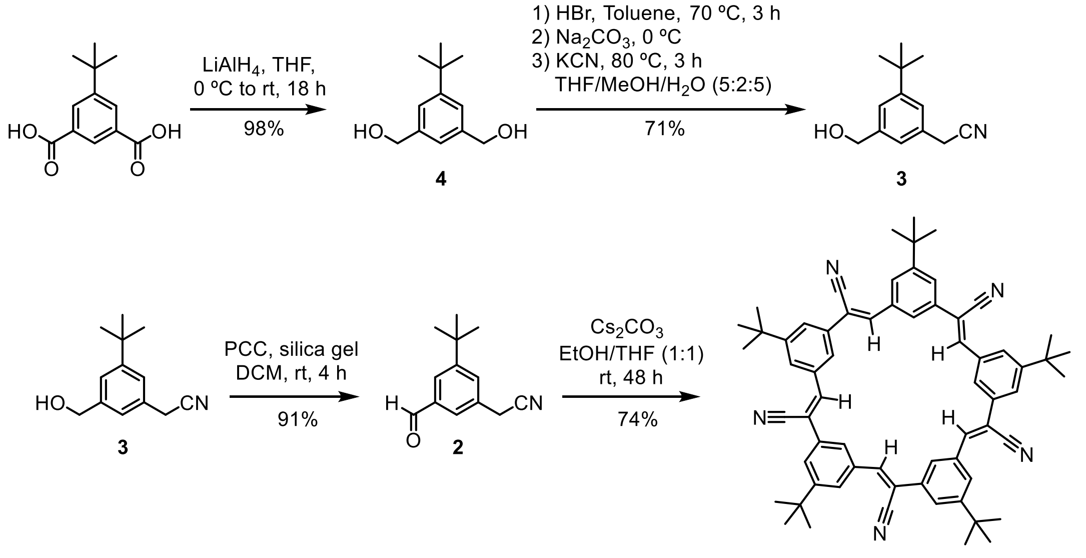

CS-1

Figure S1: Synthetic route towards CS-**1**.

Synthesis and characterization of 5-(*tert*-butyl)-1,3-dihydroxymethylenebenzene (4)

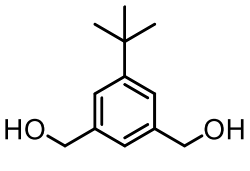

4 was prepared using a modified procedure from the one reported by Schick and co-workers.[1]

Lithium aluminium hydride (LAH) (1.02 g, 27 mmol, 6 eq.) was charged in a flame-dried 100 mL round-bottom flask and suspended in THF (freshly distilled over metallic sodium, 25 mL) under argon atmosphere and the mixture cooled to 0 °C. To this, a solution of 5-(*tert*-butyl)isophthalic acid (1.00 g, 4.5 mmol, 1 eq.) in THF (freshly distilled over

metallic sodium, 20 mL) was added dropwise, and the mixture was slowly allowed to reach room temperature and stirred under argon atmosphere for 18 h. After this time, water (15 mL) was slowly added under ice bath cooling and a saturated solution of sodium tartrate (15 mL) was subsequently added. The reaction mixture was extracted with DCM (3 × 40 mL), the organic layers were combined and dried over anhydrous $MgSO_4$, filtered and evaporated under reduced pressure to give **4** as a white solid (850 mg, 98 %). ^{1}H- and ^{13}C-NMR data were consistent with those reported in the literature.[2]

Synthesis and characterization of [3-*tert*-butyl-5-(hydroxymethyl)phenyl] acetonitrile (3)

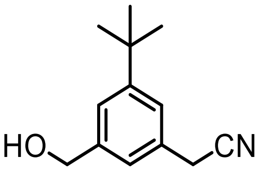

4 (500 mg, 2.57 mmol, 1 eq.) was dissolved in toluene (30 mL), heated to 70 ºC and stirred at that temperature for 10 minutes. Then, 47-49 % aqueous solution of HBr (0.38 mL, 3.34 mmol, 1.3 eq.) was added and stirred for 3 h. The reaction mixture was cooled to 0 ºC and neutralized with 15 mL of aqueous Na_2CO_3 (82 mg, 0.77 mmol, 0.3 eq.). The organic phase was collected and the aqueous layer was further extracted with AcOEt (2 × 20 mL). The organic layers were combined, dried over anhydrous $MgSO_4$, filtered and the filtrate concentrated under reduced pressure. The resulting crude product was dissolved in THF/MeOH/H_2O (5:2:5, 20 mL) and KCN (217 mg, 3.34 mmol) was added and the mixture stirred at 80 °C for 3 h. After cooling to room temperature, THF and MeOH were evaporated under reduced pressure. The mixture was extracted with AcOEt (2 × 20 mL) and the organic phases were combined, dried over anhydrous $MgSO_4$, filtered and the solvents were removed under reduced pressure. The resulting crude product was purified by column chromatography using AcOEt/*n*-heptane (1:1) as eluent, affording **3** as a pale-yellow oil (371 mg, 71 %). ^{1}H- and ^{13}C-NMR data were consistent with those reported in the literature.[3]

Synthesis and characterization of 2-(3-(*tert*-butyl)-5-formylphenyl)acetonitrile (2)

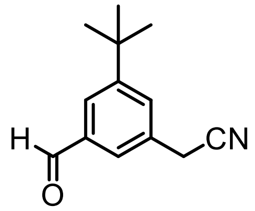

2 was prepared using a modified procedure from Lee and co-workers.[1]

Pyridinium chlorochromate (PCC, 398 mg, 1.84 mmol, 1.5 eq.) and silica gel (2.0 g) were vigorously mixed using a mortar and a pestle. Then, the mixture was loaded in a 100 mL round-bottomed flask and DCM (20 mL) was added. A solution of **3** (250 mg, 1.23 mmol, 1 eq.) in DCM (10 mL) was added to the mixture and stirred at room temperature for 4 h. The reaction mixture was directly loaded onto a short silica gel column and flushed with DCM/AcOEt (98:2) to give **2** as a clear viscous oil (225 mg, 91 %). ^{1}H- and ^{13}C-NMR data were consistent with those reported in the literature.[4]

Synthesis and characterization of cyanostar CS-1

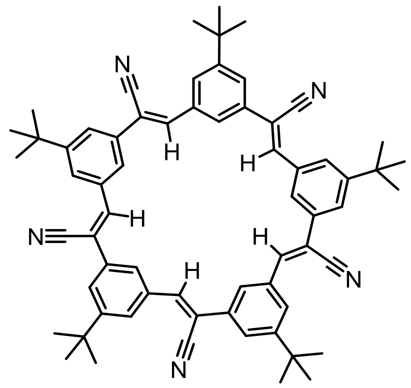

CS-**1** was prepared using a modified procedure from Lee and co-workers.[4]

A mixture of Cs_2CO_3 (19.4 mg, 0.060 mmol) and EtOH (7.5 mL) was stirred at 60 °C for 1 h. After cooling to room temperature, a solution of **2** (120 mg, 0.596 mmol) in THF (7.5 mL) was added dropwise and stirred in the dark for 48 h. After this time, the solvents were removed under vacuum. The crude solid was purified by column chromatography using a gradient from pure *n*-heptane to *n*-heptane/AcOEt (85:15). The solvents were removed under reduced pressure, and the product was recrystallized from $CHCl_3$ and Et_2O to yield CS-**1** as a white crystalline solid (81 mg, 74 %). ^{1}H- and ^{13}C-NMR data were consistent with those reported in the literature.[4]

3. Experimental methods and computational details

The scanning tunnelling microscopy (STM) experiments were performed at Fundación IMDEA-Nanoscience, in an ultra-high vacuum (UHV) setup with a base pressure of 1×10^{-10} mbar, hosting a commercial low-temperature STM from Scienta-Omicron (Polar model) held at cryogenic temperatures (4.5 K) and controlled by Nanonis MimeaBP5e electronics from SPECS. The STM images have been taken in constant current mode unless otherwise stated, with a bias voltage applied to the sample, which was held at 4.5 K.

Prior to start the experiments, the Au(111) crystal (Mateck GmbH) was prepared by repeated cycles of standard Ar^+ sputtering (1.5 keV, 10 µA, 10 min, SPECS IQE 11/35) and subsequent annealing to 450 °C during 10 min.

The cyanostar molecules were deposited on top of the clean Au substrate, held at room temperature, by organic molecular beam epitaxy from a quartz crucible held at 315 ºC (Kentax). To control the deposition, molecular flux was monitored by means of a quartz crystal microbalance (LewVac).

DFT calculations were carried out using the DMol3 package[5,6] integrated in the Material Studio program of Dassault Systèmes. The electron exchange and correlation energies were treated with the generalized gradient approximation (GGA) of Perdew, Burke, and Ernzerhof.[7] The valence electron functions were expanded to a set of numerical atomic orbitals by a double-numerical basis with polarization functions (DNP), (a polarization d function on all the non-hydrogen atoms and a polarization p function on all the hydrogen atoms). The cutoff radius was set to Rc = 4.5 Å. DFT semicore pseudopotentials (DSPP), [8] which include some degree of relativistic effects, was used for Au and Co. The Tkatchenko and Scheffler (TS) scheme[9] for dispersion correction was also included. The convergence criteria were set as follows: SCF tolerance = 2×10^{-5} eV, maximum force = 0.027 eV/Å, maximum displacement = 0.002 Å, and total energy = 2×10^{-5} eV. The Brillouin zone was sampled using a 3×3×1 Monkhorst-Pack K-point grid.[10] Due to computational limitations, the metal surface was simulated by a repeated one-layer slab that was kept frozen, with a lateral supercell

$$\begin{pmatrix} 10 & 0 \\ 6 & 12 \end{pmatrix} \text{ for the isolated molecule}$$

$$\begin{pmatrix} 7 & 1 \\ -2 & 12 \end{pmatrix} \text{ for the self-assembled layer}$$

$$\begin{pmatrix} 16 & 5 \\ 0 & 10 \end{pmatrix}$$ for the (CS-**1**)···Co···(CS-**1**) complex

and a unit cell length in the perpendicular direction of 50 Å, in order to avoid the interaction between adjacent structures. Although the thickness of the gold layer can be considered small to study electronic effects, it is enough to induce an adsorption geometry in agreement with the experimental results.

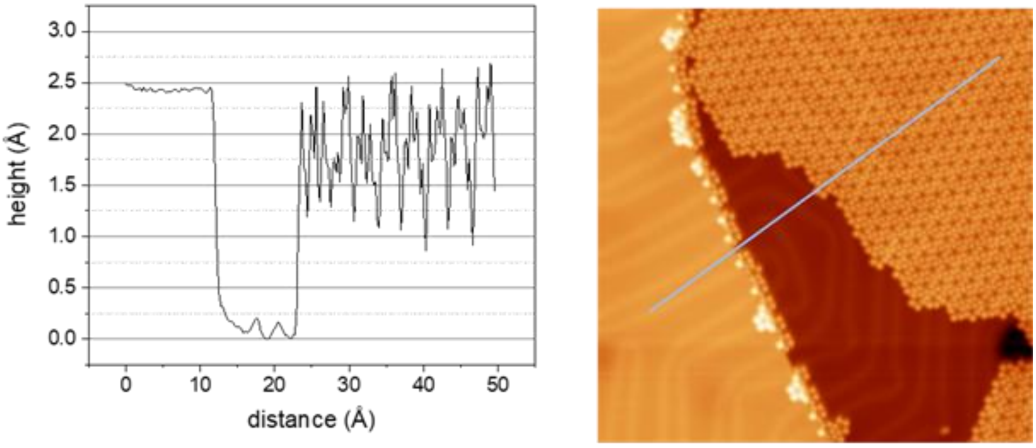

Figure S2: a) Height line profile along the line drawn in the b) STM image (10 mV, 10 pA, 50 nm × 50 nm), that crosses a Au step (with a nominal height of 2.36 Å).

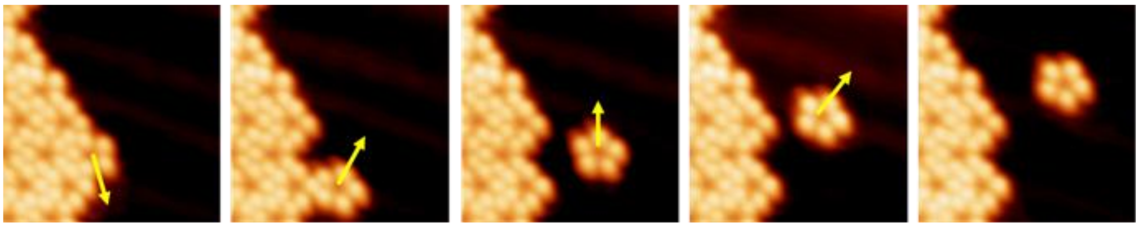

Figure S3: Sequence of high resolution STM images (100 mV, 10 pA, 10 nm × 10 nm) taken after applying a lateral manipulation procedure to the macrocycle on the left-hand side image. By approaching the tip to the surface (10 mV, 120 pA), tracing a line over the molecule, and retracing back the tip to normal imaging conditions, the translation of the cyanostar molecule on the Au(111) surface can be observed.

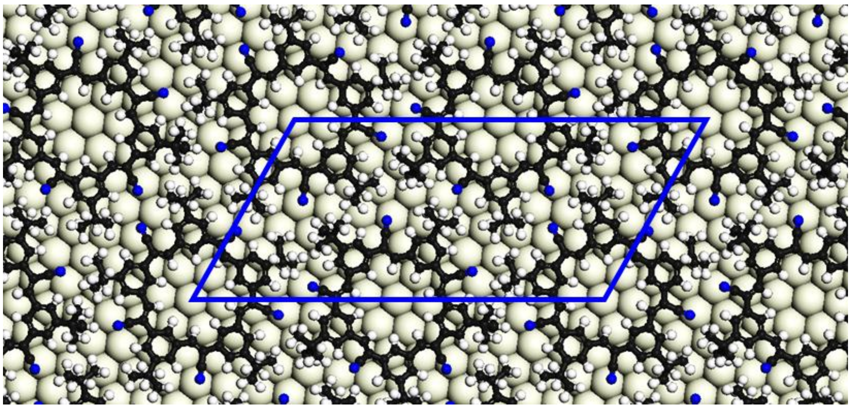

Figure S4: DFT optimized structure of the self-assembly of CS-**1** on Au(111). The blue rhomboid indicates the unit cell of the self-assembly. Carbon atoms are coloured in black, nitrogen atoms in blue, hydrogen atoms in white, and gold atoms in light grey.

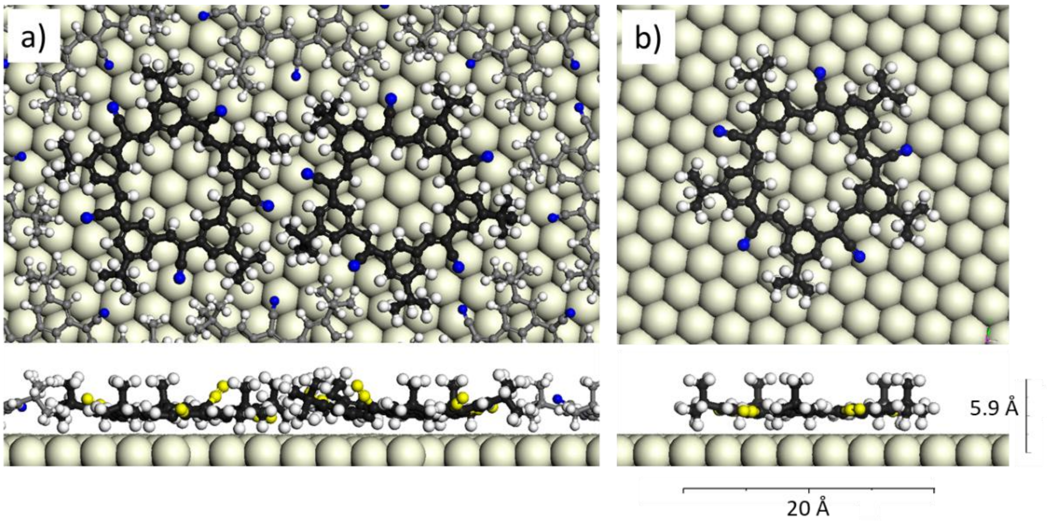

Figure S5: a) Top and side view of the DFT optimized self-assembled structure of the cyanostar molecule on the Au(111) surface. For clarity, the two molecules forming the unit lattice are singled-out. In the bottom panel the cyano groups are drawn in yellow, to help in visualizing their inclination with respect to the Au(111) surface. b) Same as a) but for an isolated molecule on Au(111), with scale bars indicating the molecular dimensions on the surface. Carbon atoms are coloured in black, nitrogen atoms in blue, hydrogen atoms in white, and gold atoms in light grey.

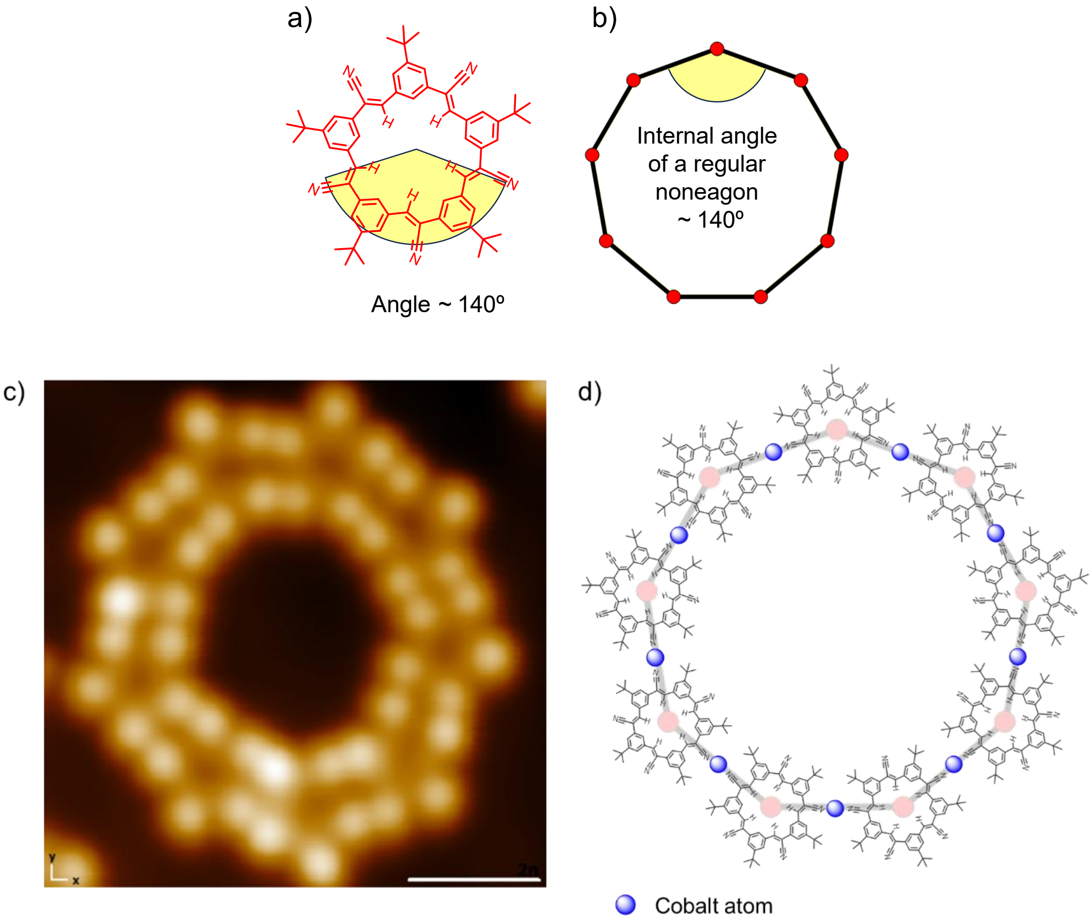

Figure S6: a) Chemical structure of CS-**1** superimposed to a slice geometrical figure with an internal angle of 140º. b) Schematic representation of a regular noneagon featuring internal angles of 140º. c) STM image of a circular ensemble composed of 9 cyanostar molecules coordinated through Co atoms (same STM figure as in Figure 4e). d) Proposed model of the circular ensemble of Figure S6c composed of 9 cyanostars "bridged" by Co atoms (blue dots). Underneath the metalo-organic complex, a regular noneagon has been depicted.

References

[1] T. H. G. Schick, F. Rominger, M. Mastalerz, *J. Org. Chem.*, **2020**, *85*, 13757–13771.

[2] S. H. Hwang, C. N. Moorefield, L. Dai, G. R. Newkome, *Chem. Mater.*, **2006**, *18*, 4019–4024.

[3] E. Alcalde, C. Ayala, I. Dinarès, N. Mesquida, *J. Org. Chem.*, **2001**, *66*, 2291–2295.

[4] S. Lee, C. H. Chen, A. H. Flood, *Nat. Chem.*, **2013**, *5*, 704–710.

[5] B. Delley, *J. Chem. Phys.*, **1990**, *92*, 508–517.

[6] B. Delley, *J. Chem. Phys.*, **2000**, *113*, 7756–7764.

[7] J. P. Perdew, K. Burke, M. Ernzerhof, *Phys. Rev. Lett.*, **1996**, *77*, 3865–3868.

[8] B. Delley, *Phys. Rev. B*, **2002**, *66*, 155125.

[9] A. Tkatchenko and M. Scheffler, *Phys. Rev. Lett.*, **2009**, *102*, 073005.

[10] H. J. Monkhorst and J. D. Pack, *Phys. Rev. B*, **1976**, *13*, 5188–5192.
